# Synergistic antifibrotic effects of miR-451 with miR-185 partly by co-targeting EphB2 on hepatic stellate cells

**DOI:** 10.1038/s41419-020-2613-y

**Published:** 2020-05-28

**Authors:** Xiaogang Chen, Dan Zhang, Yi Wang, Ke Chen, Limeng Zhao, Yating Xu, Hulin Jiang, Shuzhen Wang

**Affiliations:** 1 State Key Laboratory of Natural Medicines, School of Life Science and Technology, China Pharmaceutical University, 211198 Nanjing, China; 2 State Key Laboratory of Natural Medicines, Department of Pharmaceutics, China Pharmaceutical University, 211198 Nanjing, China

**Keywords:** Target validation, Experimental models of disease

## Abstract

Liver fibrosis is a global health problem currently without clinically approved drugs. It is characterized by the excessive accumulation of extracellular matrix (ECM) mainly produced by activated hepatic stellate cells (HSCs). Uncovering the mechanisms underlying the fibrogenic responses in HSCs may have profound translational implications. Erythropoietin-producing hepatocellular receptor B2 (EphB2) is a receptor tyrosine kinase that has been indicated to be a novel profibrotic factor involved in liver fibrogenesis. In the present study, we investigated the effects of miR-451 and miR-185 on the expression of EphB2 and their roles in liver fibrogenesis both in vitro and in vivo. We found that EphB2 upregulation is a direct downstream molecular event of decreased expression of miR-451 and miR-185 in the process of liver fibrosis. Moreover, miR-451 was unexpectedly found to upregulate miR-185 expression at the post-transcriptional level by directly targeting the nuclear export receptor exportin 1 (XPO-1) and synergistically suppress HSCs activation with miR-185. To investigate the clinical potential of these miRNAs, miR-451/miR-185 agomirs were injected individually or jointly into CCl_4_-treated mice. The results showed that coadministration of these agomirs synergistically alleviated liver fibrosis in vivo. These findings indicate that miR-451 and miR-451/XPO-1/miR-185 axis play important and synergistic regulatory roles in hepatic fibrosis partly through co-targeting EphB2, which provides a novel therapeutic strategy for the treatment of hepatic fibrosis.

## Introduction

Liver fibrosis is a dynamic wound-healing process accompanied by the excessive accumulation of extracellular matrix (ECM) as well as scar formation arising from various chronic liver diseases, such as viral infection, alcoholic liver disease (ALD) and nonalcoholic steatohepatitis (NASH)^1^. Long-term liver fibrosis results in progressive loss of liver function, cirrhosis, and even hepatocellular carcinoma (HCC)^2,3^. In this process, quiescent hepatic stellate cells (HSCs) are activated in response to liver injury and transforming to myofibroblast-like HSCs, the major producer of excess ECM, which are pivotal steps in the initiation and progression of liver fibrosis^4,5^. Nevertheless, due to the remarkable complexity and irreversible trans-differentiation of HSCs activation, its underlying mechanism and complex regulatory networks are still unclear, which leads to the limited treatment options for liver fibrosis. Therefore, clarifying its regulation is important for advancing the development of effective antifibrotic therapies.

To date, growing numbers of studies have shown that aberrant activity of receptor tyrosine kinases (RTKs) participated in multiple fibrotic diseases including lung fibrosis, kidney fibrosis, and liver fibrosis^6–8^. As one of the largest family of RTKs, EphB2 is initially identified as an axon guidance cue and plays a well-defined role in synaptic plasticity and central nervous system development^9,10^. While subsequent researches revealed that it is also universally expressed in various non-neural cells and modulates diverse biological processes including tumor formation and liver metastasis^11–13^. Recently, several studies reported that EphB2 was abnormally upregulated in mice model of liver fibrosis induced by infection or inflammation, and could be potentially targeted for novel antifibrotic therapies^14–16^. However, the detailed mechanism for the upregulation of EphB2 in liver fibrosis remains to be fully elucidated.

MicroRNAs (miRNAs) are conserved, endogenous, small noncoding RNAs that repress gene expression through its seed sequences partly or completely binding the 3′ untranslated region (3′ UTR) of mRNA transcripts in the cytoplasm^17,18^, or serve as an enhancer to trigger activating gene transcription by modifying chromatin status in the nucleus^19,20^. Because of their gene-regulating ability, miRNAs play regulatory roles in diverse hepatic biological and pathological processes, such as HSCs activation, development of hepatic fibrosis and HCC^21,22^, and could be developed into novel therapies to reverse the progression of hepatic fibrosis. After an extensive literature review and bioinformatic analysis using available miRNA target-prediction tools online, such as miRbase, miRanda and miRTarBase, we hypothesized that the aberrantly expressed EphB2 might correlate with two liver disease-related miRNAs including miR-451 and miR-185 to drive fibrogenesis.

In this study, we investigated whether miR-451/miR-185 is related to liver fibrosis, and probed the relationship between these miRNAs and EphB2 gene. We found that the activation of HSCs was associated with downregulation of miR-451/miR-185 accompanied by upregulation of EphB2 and other fibrosis markers, which were further confirmed in CCl_4_-induced hepatic fibrosis models. EphB2 was then confirmed to be a target gene of these miRNAs. Moreover, our results revealed that overexpression of miR-451 could upregulate miR-185 expression at the post-transcriptional level through repressing the expression of the nuclear export receptor XPO-1 (also known as chromosome region maintenance 1, CRM1). Therefore, the synergic antifibrotic actions between miR-451 and miR-185 were then tested and verified in HSCs and CCl_4_-treated mice. These proof-of-concept results further delineate a novel mechanism for upregulation of EphB2 in liver fibrosis and provide a potential therapeutic strategy for treatment of liver fibrosis.

## Materials and methods

### Cell line and isolation of primary HSCs

The immortalized human HSCs LX-2 and rat HSCs HSC-T6 were purchased from Xiangya School of Medicine (Hunan, China) and the Chinese Academy of Sciences Shanghai Institute of Cell Bank (Shanghai, China), respectively. HEK 293T cells were obtained from KeyGEN Biotech (Nanjing, China). All the cells were maintained in Dulbecco’s modified Eagle’s medium (DMEM) (KeyGEN Biotech, China) containing 10% fetal bovine serum (FBS) (Biological Industries, Israel), and incubated at 37 °C and 5.0% CO_2_. All cell lines were authenticated by using short tandem repeat matching analysis. No mycoplasma contamination was detected.

Primary HSCs were isolated from normal male C57BL/6 mice at 7−8 weeks of age by in situ perfusion with digestive enzymes (Sigma, USA) and Density gradient centrifugation as described previously^23^. The purity of the obtained HSCs was more than 95%. The isolated HSCs were maintained in DMEM supplemented with 20% FBS and incubated at 37 °C in a 5% CO_2_ humidified incubator.

### RNA extraction and RT-qPCR

Total RNAs were isolated from cells or liver tissues using TRIzol reagent (Invitrogen, Carlsbad, CA, USA). The concentration and purity of RNA were detected by Nanodrop spectrophotometer. cDNA was synthesized using Reverse Transcription Kit (Vazyme Biotech, Nanjing, China), and RT-qPCR was performed using SYBR Green Master Mix (Vazyme Biotech, Nanjing, China). The relative mRNA level was normalized to the GAPDH gene. 2^−△△Ct^ relative quantitative method was used to evaluate gene expression. The primer sequences used in this study are listed in Supplementary Table 1.

For miRNA analysis, the first-strand cDNA was synthesized using reverse transcriptase with a miRNA-specific stem-loop primer (RiboBio, Guangzhou, China) and RT-qPCR analysis was employed using the Bulge-Loop miRNA RT-qPCR Starter Kit (RiboBio, Guangzhou, China). The relative expression level of miRNA was normalized to U6 gene by using the 2^−△△Ct^ method.

### Western blotting

Protein was extracted from cells and liver tissues with lysis buffer (Beyotime Biotechnology, Shanghai, China), and its concentration was quantified by the BCA Assay Kit (Generay Biotech, Shanghai, China). Equal amount of proteins were separated by sodium dodecyl sulfate-polyacrylamide gel electrophoresis and then transferred onto polyvinylidene fluoride membrane (Millipore, Billerica, MA, USA). After blocking with 5% non-fat milk in TBST for 2 h at room temperature, the membranes were incubated overnight with specific primary antibodies at 4 °C. The membranes were then incubated with secondary antibodies for 2 h at room temperature and eventually visualized using ECL Western blotting substrate (Millipore, Billerica, MA, USA). Antibodies used in this study were as follows: rabbit anti-EphB2 (D2X2I; Cell Signaling Technologies, Danvers, MA, USA), rabbit anti-MMP2 (D8N9Y; Cell Signaling Technologies, Danvers, MA, USA), rabbit anti-α-SMA (ab32575; Abcam, Cambridge, UK), rabbit anti-TIMP2 (D18B7; Cell Signaling Technologies, Danvers, MA, USA), mouse anti-XPO-1 (66763-1-lg; Proteintech, Wuhan, China), rabbit anti-RHEB (15924-1-AP; Proteintech, Wuhan, China), mouse anti-RICTOR (220462; ZEN BIO, Chengdu, China), rabbit anti-c-myc (9402S; Cell Signaling Technologies, Danvers, MA, USA), mouse anti-GAPDH (60004-1-lg; proteintech, Wuhan, China), rabbit anti-Tubulin (10094-1-AP; proteintech, Wuhan, China), anti-rabbit IgG, HRP-linked antibody (7074S; Cell Signaling Technologies, Danvers, MA, USA), and anti-mouse IgG, HRP-linked antibody (7076S; Cell Signaling Technologies, Danvers, MA, USA).

### Cell transfection and treatment

HSCs were transfected with miRNAs mimics, inhibitor or negative control (NC) targeting human EphB2 mRNA (GenePharma, Shanghai, China) at a final concentration of 100 nM using Lipofectamine 2000 transfection reagent (Invitrogen, Carlsbad, CA, USA). After 24 or 48 h, cells were harvested for RT-qPCR or Western blotting analysis. To study the effect of XPO-1 on the regulation of miR-185 expression, HSCs were treated with 0.5 or 1.0 μM of XPO-1 inhibitor Verdinexor (CSN17781, CSNpharm, USA) for 48 h.

### Plasmid construction and luciferase reporter assay

The wild-type or mutant type 3′ untranslated region (3′UTR region) of EphB2 or XPO-1 gene were cloned into pmir-GLO dual-luciferase miRNA Target Expression Vector (Miaolingbio, Wuhan, China), respectively. HEK 293T cells were then cotransfected with the plasmid constructed above and miRNA mimics using Lipofectamine 2000. After 48 h, cells were collected, lysed and luciferase activities were measured using Double Luciferase Reporter Gene Assay Kit (Beyotime Biotechnology, Shanghai, China). The firefly luciferase activities were normalized to Renilla luciferase activity.

### Animal studies

Male C57BL/6 mice (6−8 weeks, 18−22 g) were purchased from SPF Biotechnology Co., Ltd (Beijing, China), and raised at the pharmaceutical animal experimental center of China Pharmaceutical University. All animal experiments were performed in accordance with the National Institutes of Health Guide for the Care and Use of Laboratory Animals, with the approval of the Animal Experimentation Ethics Committee of China Pharmaceutical University.

For CCl_4_-induced liver fibrosis model, mice were injected intraperitoneally with 0.1 mL of corn oil or CCl_4_/corn oil (1:9, v/v)/10 g body weight twice a week for 4 weeks. To investigate the effects of miR-451 and miR-185 on hepatic fibrosis in vivo, a modified protocol based on the described method by Ma et al.^24^ was employed. Mice were randomly divided into four experimental groups: CCl_4_/agomir negative control (NC) group, CCl_4_/agomiR-451 group, CCl_4_/agomiR-185 group, and CCl_4_/agomiR-451/agomiR-185 group. AgomiRNAs (GenePharma, Shanghai, China) were packaged within hyperbranched lipoid-based lipid nanoparticles (VLNPs) to obtain miRNA-loaded nanoparticles as described previously^25^. Four weeks of treatment started after 2 weeks of CCl_4_ injection; the mice were administered with agomiRNAs (1 mg/kg body weight) via tail vein twice a week at the next day of CCl_4_ injection. All mice were sacrificed 48 h after the last injection to collect the serum and liver tissues for subsequent analysis.

### Hydroxyproline assay

To assess the liver function, 30−50 mg of wet liver tissues were subjected to acid hydrolysis, and the content of hydroxyproline was determined according to the manufacturer’s protocol of Hydroxyproline Testing Kit (Nanjing Jiancheng Bioengineering Institute, China).

### Assessment of liver fibrosis

The tissues were fixed with 4% paraformaldehyde, and paraffin sections were prepared. They were stained with hematoxylin and eosin (H&E) staining and Masson Staining Kit (Servicebio Technology, Wuhan, China). Mice blood samples were obtained, and the serum levels of T-bilirubin (T-bil), alanine transaminase (ALT) and aspartate transaminase (AST) were measured by Total Bilirubin Assay Kit, Alanine Aminotransferase Assay Kit and Aspartate Aminotransferase Assay Kit (Servicebio Technology, Wuhan, China), respectively.

### Statistical analysis

The data are shown as the mean ± SEM from at least three independent experiments. The differences between the groups were analyzed using Student’s *t* test. Analyses were performed using the GraphPad Prism program (version 7.0; San Diego, CA, USA). All statistical tests were two-sided, and *P* < 0.05 was considered statistically significant.

## Results

### Expression of miR-451/miR-185 is downregulated in activated HSCs

**Fig. 1:**
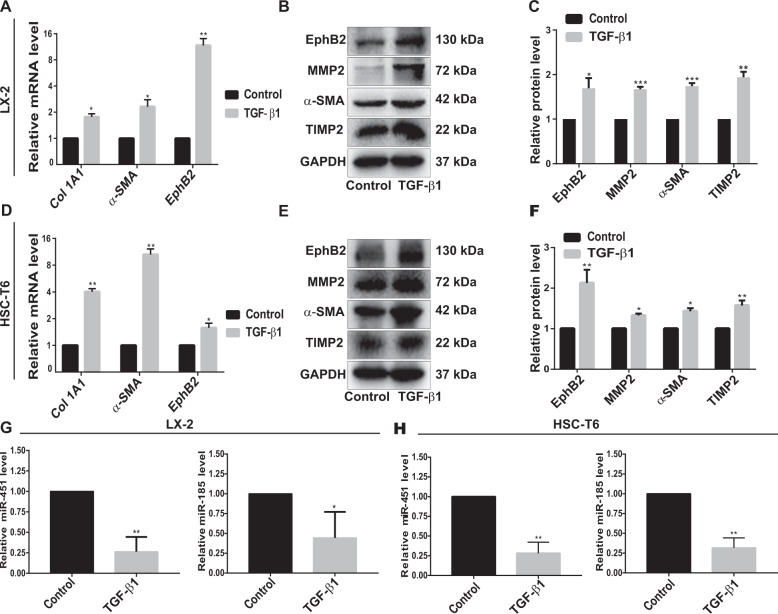
Expression of HSCs activation-related genes and miR-451/miR-185 in activated HSC cell lines. **a** The mRNA levels of *Col 1A1, α-SMA* and *EphB2* were examined in LX-2 cells using RT-qPCR. **b** The protein expressions of EphB2, MMP2, α-SMA and TIMP2 were analyzed in LX-2 cells using western blotting. **c** Protein bands in (**b**) were quantified by ImageJ software. **d** The mRNA levels of *Col 1A1*, *α-SMA* and *EphB2* were examined in HSC-T6 cells using RT-qPCR. **e** The protein expressions of EphB2, MMP2, α-SMA and TIMP2 were analyzed in HSC-T6 cells using western blotting. **f** Protein bands in (**e**) were quantified by ImageJ software. **g** The expression of miR-451/miR-185 was measured in LX-2 cells by RT-qPCR. **h** The expression of miR-451/miR-185 was measured in HSC-T6 cells by RT-qPCR. Data represent the means ± SEM obtained from triplicate experiments (Student’s *t* test, *P < 0.05, **P < 0.01, and ***P < 0.001).

After transforming growth factor β1 (TGF-β1) stimulation, the mRNA and protein levels of HSCs activation-related markers including Collagen Type I Alpha 1 Chain (Col 1A1), α-smooth muscle actin (α-SMA), matrix metallopeptidase 2 (MMP2) and tissue inhibitor of metalloproteinase 2 (TIMP2) were significantly upregulated in human LX-2 cells (Fig. 1a–c). Similar results were also obtained in rat HSC-T6 cells (Fig. 1d–f). Meanwhile, a significantly increased mRNA and protein expression of EphB2 was observed during the process of HSCs activation (Fig. 1a–f). The results also showed that the expression of miR-451/miR-185 was markedly downregulated by TGF-β1 treatment in both LX-2 and HSC-T6 cells as compared with the control group (Fig. 1g, h), suggesting that these miRNAs might play important roles in HSCs activation.

### Expression of miR-451/miR-185 is reduced during primary HSCs activation

**Fig. 2:**
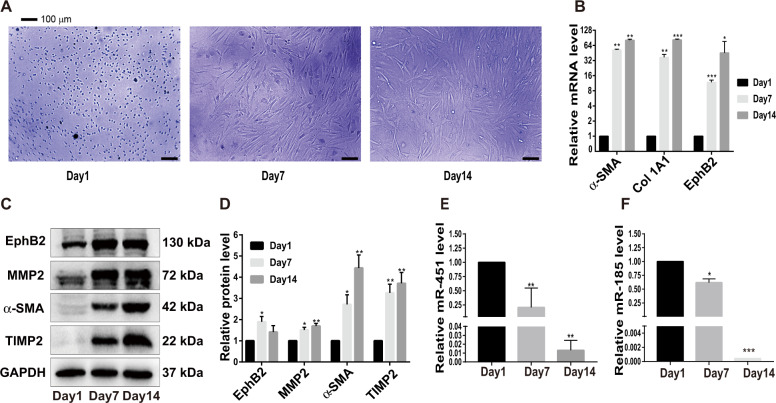
Expression of HSCs activation-related genes and miR-451/miR-185 in activated primary HSCs. **a** The morphological changes of mouse primary HSCs on the 1st, 7th and 14th day of culture in vitro at ×100 magnification (scale bar, 100 μm). **b** RT-qPCR analysis of *α-SMA*, *Col 1A1* and *EphB2* mRNA levels in primary HSCs. **c** Western blotting analysis of EphB2, MMP2, α-SMA and TIMP2 in primary HSCs. **d** Protein bands in (**c**) were quantified by ImageJ software. **e**, **f** The expression of miR-451 and miR-185 was measured in primary HSCs by RT-qPCR. Data represent the means ± SEM obtained from triplicate experiments (Student’s *t* test, *P < 0.05, **P < 0.01, and ***P < 0.001).

To examine changes in the expression of miR-451/miR-185 during the trans-differentiation of HSCs from quiescent state to activated phenotype, we isolated primary HSCs from C57BL/6 mice. After cultured in vitro for 14 days, the primary HSCs were activated in a time-dependent manner and acquired distinctive astral-like morphology (Fig. 2a). The activated HSCs exhibited significant increases in mRNA levels of Col 1A1and α-SMA (Fig. 2b) and protein levels of MMP2, α-SMA and TIMP2 (Fig. 2c, d). The mRNA and protein expression levels of EphB2 were also noticeably increased during the process of HSCs activation (Fig. 2b–d). At day 14, the mRNA levels of miR-451 and miR-185 were sharply reduced (Fig. 2e, f), as evidenced in LX-2 and HSC-6 cell lines. These results showed that the expression of miR-451 and miR-185 was significantly inhibited during primary HSCs activation.

### Expression of miR-451/miR-185 is repressed in CCl_4_-induced hepatic fibrosis

**Fig. 3:**
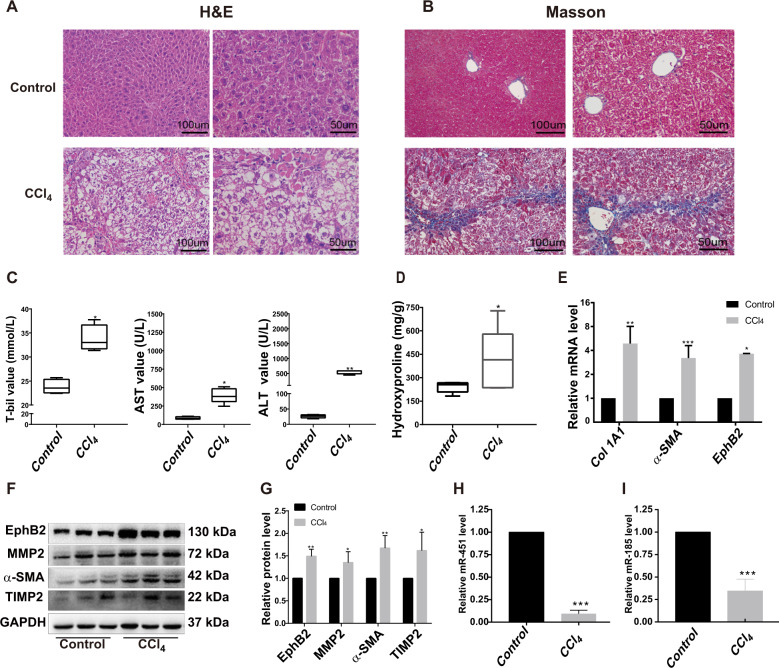
Expression of HSCs activation-related genes and miR-451/miR-185 in CCl_4_-induced mice. **a**, **b** Representative histopathological images of H&E staining and Masson’s trichrome staining of mouse liver sections at ×200 magnification and ×400 magnification (scale bar, 50 μm or 100 μm). **c** The levels of AST, ALT, T-bil in the serum of mice treated with oil or CCl_4_ at 4 weeks. **d** Analysis of liver hydroxyproline content using hydroxyproline assay kit. **e** RT-qPCR analysis for the expression of *EphB2*, *α-SMA*, and *Col 1A1* in liver tissues of the oil or CCl_4_-treated mice at 4 weeks. **f** Western blotting analysis for the protein expression of EphB2, MMP2, α-SMA and TIMP2 in hepatic tissues of representative mice from each group (*n* = 6 per group). **g** Proteins bands in (**f**) were quantified by ImageJ software. **h**, **i** The expression of miR-451 and miR-185 in CCl_4_-treated mice was examined by RT-qPCR. Data are shown as the means ± SEM obtained from triplicate experiments (Student’s *t* test, *P < 0.05, **P < 0.01, and ***P < 0.001).

The widely used CCl_4_-induced liver fibrosis model was then employed to further examine the expression of miR-451/miR-185 during the progression of liver fibrosis. The successful establishment of the liver fibrosis model was confirmed from the histological analysis of liver tissues and blood serum biochemical analysis. H&E and Masson’s trichrome staining clearly revealed the presence of liver fibrosis, such as hepatocyte apoptosis and collagen deposition (Fig. 3a, b). Serum total T-bil, ALT, AST and hydroxyproline significantly were increased in CCl_4_-treated mice (Fig. 3c, d). Moreover, the expressions of the key profibrotic marker genes were significantly enhanced as shown by RT-qPCR (Fig. 3e) and Western blotting (Fig. 3f, g). As expected, the mRNA and protein levels of EphB2 were also statistically higher in fibrotic liver (Fig. 3e, f). In addition, examination of the expression of miR-451/miR-185 revealed between 70 and 90% downregulation in fibrotic livers (Fig. 3h, i). Taken together, these results verified the profibrotic role of EphB2, demonstrated a reduced expression of miR-451/miR-185 during the progression of liver fibrosis, and also revealed a negative relationship between EphB2 and miR-451/miR-185 expression.

### EphB2 is a downstream target of miR-451/miR-185

**Fig. 4:**
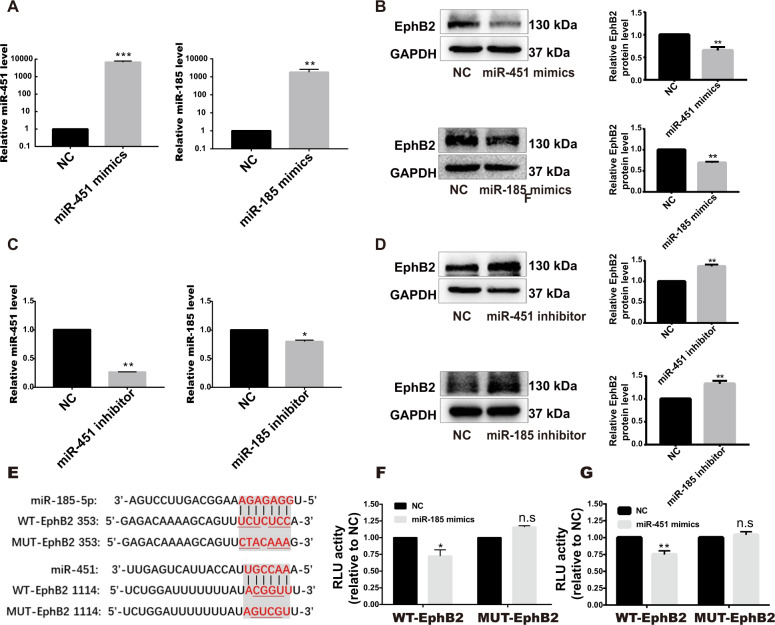
EphB2 is a target of miR-451 and miR-185. **a** Expression of miR-451/miR-185 was examined by RT-qPCR in LX-2 cells transfected with corresponding miRNA mimics. **b** Western blotting analysis for EphB2 in LX-2 cells transfected with miR-451/miR-185 mimics. **c** Expression of miR-451/miR-185 was examined by RT-qPCR in LX-2 cells transfected with corresponding miRNA inhibitor. **d** Western blotting analysis for EphB2 in LX-2 cells transfected with miR-451/miR-185 inhibitor. **e** Potential binding sites (red font) for miR-451/miR-185 in the 3′UTR of *EphB2* mRNA. **f**, **g** Dual luciferase reporter assay showed miR-451/miR-185 mimics could inhibit the luciferase activity with wild-type 3′UTR of EphB2 mRNA but had no significant influence on that with mutant 3′UTR, suggesting EphB2 is a direct target of miR-451/miR-185. Data are shown as the means ± SEM obtained from triplicate experiments (Student’s *t* test, *P < 0.05, **P < 0.01, and ***P < 0.001, n.s. nonsignificant).

To probe the target relationship of miR-451/miR-185 with EphB2 during the activation of HSCs, synthetic miR-451/miR-185 mimics or inhibitor were transiently transfected into LX-2 or HSC-T6 cells after TGF-β1 stimulation. As shown in Fig. 4a, b, the protein expression of EphB2 was markedly reduced by overexpression of miR-451/miR-185. Correspondingly, treatment with miR-451/miR-185 inhibitor significantly enhanced the expression of EphB2 (Fig. 4c, d). Similar results were also observed in HSC-T6 cells transfected with miR-451/miR-185 mimics or inhibitor (Supplementary Fig. S1). Through miRNA target gene prediction website including microrna.org and miRBase, we obtained the potential binding sites between miR-451/miR-185 and *EphB2* mRNA (Fig. 4e). We cloned the wild-type or mutant 3′UTR of *EphB2* mRNA into the dual-luciferase reporter vector, and cotransfected each vector with miR-451/miR-185 mimics or scramble control, respectively. The results showed that the luciferase activities were significantly decreased in cells cotransfected with miR-451/miR-185 mimics with wild-type 3′UTR of *EphB2* mRNA, confirming that EphB2 is a novel direct target of these miRNAs in HSC cells (Fig. 4f, g).

### MiR-451 combined with miR-185 synergistically suppresses EphB2 expression and HSCs activation in vitro

**Fig. 5:**
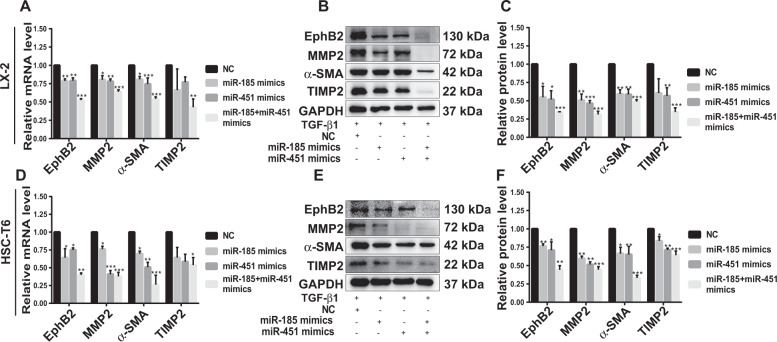
MiR-451 and miR-185 synergistically suppress HSCs activation in vitro. **a** RT-qPCR analysis of the mRNA levels of *MMP2*, *α-SMA*, and *TIMP2* in LX-2 cells transfected with miR-451 mimics, miR-185 mimics, their combinations or control. **b**, **c** Western blotting analysis of EphB2 and MMP2, α-SMA, and TIMP2 in LX-2 cells transfected with miR-451 mimics, miR-185 mimics, their combinations or control. **d** RT-qPCR analysis of the mRNA levels of *MMP2*, *α-SMA*, and *TIMP2* in HSC-T6 cells transfected with miR-451 mimics, miR-185 mimics, their combinations or control. **e**, **f** Western blotting analysis of EphB2, MMP2, α-SMA and TIMP2 in HSC-T6 cells transfected with miR-451 mimics, miR-185 mimics, their combinations or control. Data are shown as the means ± SEM obtained from triplicate experiments (Student’s *t* test, *P < 0.05, **P < 0.01, and ***P < 0.001).

Given that both miR-451 and miR-185 could bind to the 3′UTR of *EphB2* mRNA and downregulate EphB2 protein expression, we next examined the collaborative functions of these miRNAs in HSCs cells. LX-2 and HSC-T6 cells were transfected with NC, miR-185 mimics, miR-451 mimics or the combination of both at half dose. Our results showed that miR-451 and miR-185 mimics caused significant reduction in the mRNA and protein levels of EphB2, MMP2, α-SMA and TIMP2 in both LX-2 and HSC-T6 cells, respectively (Fig. 5). This demonstrated that miR-451 and miR-185 play inhibitory roles in liver fibrogenesis, which was also supported by the finding that miR-451/miR-185 downregulation by inhibitor reversed its inhibitory effect on liver fibrosis (Supplementary Fig. S2). Moreover, combination of half-dose miR-451 and half-dose miR-185 presented better inhibitory effect on the expression of EphB2 and other fibrotic markers compared with single miRNA treatment at full dose (Fig. 5), which suggested that these two miRNAs could work respectively as well as synergistically in the suppression of HSCs activation.

### MiR-451 promotes miR-185 maturation at the post-transcriptional level by directly targeting XPO-1 in HSCs

**Fig. 6:**
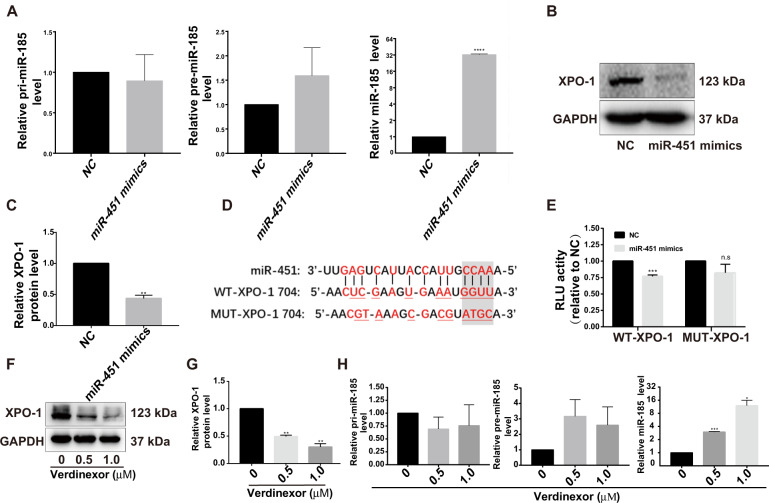
MiR-451 upregulates miR-185 at the post-transcriptional level by directly targeting XPO-1 in LX-2 cells. **a** RT-qPCR analysis of pri-miR-185, pre-miR-185 and miR-185. **b**, **c** Western blotting analysis of XPO-1. **d** Putative binding sites (red font) of miR-451 were predicted in the 3′UTR of *XPO-1* mRNA. The vertical line represents complementary base pairs between miR-451 and XPO-1 mRNA, and the gray shading indicates the seed sequence of miR-451. **e** Dual luciferase reporter assay was performed to examine the binding between miR-451 and the 3′UTR of *XPO-1* mRNA. HEK 293T cells were cotransfected with pmirGLO vectors containing either the wild-type (WT) or mutant (MUT) target sites plus either miR-451 mimics or the negative control. **f**, **g** Western blotting analysis of the expression of XPO-1 in LX-2 cells treated with XPO-1 inhibitor, verdinexor. **h** The expression of pri-miR-185, pre-miR-185 and miR-185 was determined by RT-qPCR in LX-2 cells treated with verdinexor. Data are shown as the means ± SEM obtained from triplicate experiments (Student’s *t* test, *P < 0.05, **P < 0.01, ***P < 0.001, and ****P < 0.0001, n.s. nonsignificant).

We next explored the synergistic mechanisms between miR-451 and miR-185. It was accidently observed that miR-451 overexpression resulted in about 30-fold/12-fold increase in mature miR-185, in LX-2 cells (Fig. 6a)/HSC-T6 cells (Supplementary Fig. S3A), but miR-185 showed no such effect on the expression of miR-451 (Supplementary Fig. S3B). However, no significant changes in pri-miR-185 and pre-miR-185 were detected (Fig. 6a, Supplementary Fig. S3A), which indicated that miR-451 may upregulate the biogenesis of miR-185 at the post-transcriptional level by targeting other genes. Further bioinformatics and microRNA biogenesis pathway analysis suggested that XPO-1, a nuclear-cytoplasmic transporter^26^, might be a target of miR-451. To evaluate our speculation, the upregulation of XPO-1 was firstly examined and confirmed in TGF-β1-stimulated LX-2, HSC-T6 cells and CCL_4_-induced mice (Supplementary Fig. S4). We then treated HSCs with miR-451 mimics. Our data showed that miR-451 upregulation significantly inhibited the expression of XPO-1 (Fig. 6b, c, Supplementary Fig. S3C, S3D). Using dual-luciferase reporter assay, we confirmed that miR-451 targeted XPO-1 at a conserved seed region in its 3′UTR (Fig. 6d, e). To further determine the role of XPO-1 in the expression of miR-185, we treated HSCs with a small molecule selective inhibitor of XPO-1 verdinexor at a dose of 0.5 or 1.0 μM for 48 h. As shown by western blotting, XPO-1 protein level was successfully reduced (Fig. 6f, g, Supplementary Fig. S3E, S3F) and mature miR-185 rather than pri-miR-185 and pre-miR-185 was significantly upregulated as expected (Fig. 6h, Supplementary Fig. S3G). Correspondingly, inhibiting XPO-1 by verdinexor also inhibited the expression of EphB2 as expected (Supplementary Fig. S5). Taken together, we demonstrated that XPO-1 inhibition by miR-451 led to upregulation of miR-185 at the post-transcriptional level in HSCs.

### MiR-451 combined with miR-185 synergistically attenuates liver fibrosis in vivo

**Fig. 7:**
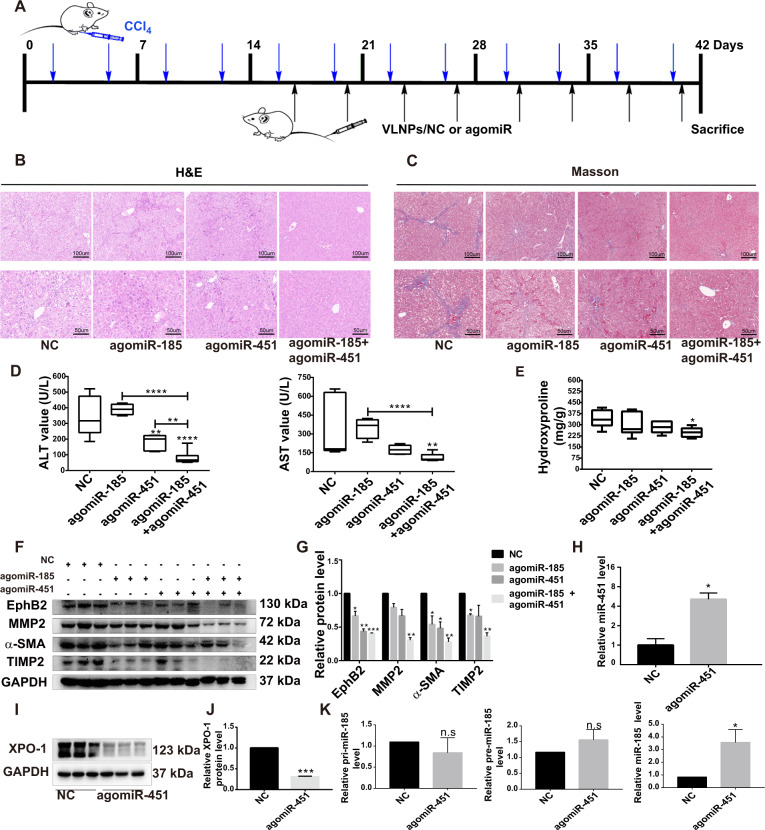
MiR-451 combined with miR-185 synergistically attenuates liver fibrosis in vivo. **a** Schema of the mice experimental protocol. **b**, **c** Representative histopathological images of H&E staining, Masson’s trichrome staining of mouse liver sections at ×200 magnification (scale bar, 50 μm) and ×400 magnification (scale bar, 100 μm). **d** AST and ALT levels in the serum of the mice treated with NC and agomiRNA. **e** Hydroxyproline content in the livers of mice treated with NC and agomiRNA. **f** Western blotting analysis for the expressions of EphB2, MMP2, α-SMA and TIMP2 in hepatic tissues of representative mice from each group (*n* = 6 per group), and **g** the intensity of the protein bands was quantified by ImageJ software. **h** RT-qPCR analysis of miR-451 in mice administrated with agomiR-451. **i** The expression of XPO-1 was decreased by agomiR-451 through western blotting analysis, and **j** quantified by ImageJ software. **k** RT-qPCR analysis for the expression of pri-miR-185, pre-miR-185, and miR-185 in livers from mice treated with NC and agomiR-451. Data are shown as the means ± SEM (Student’s *t* test, *P < 0.05, **P < 0.01, ***P < 0.001, and ****P < 0.0001, n.s. nonsignificant).

To explore the role of miR-451 and miR-185 in the prevention or treatment potential of liver fibrosis in vivo, we administrated VLNPs/NC, VLNPs/agomiR-451, VLNPs/agomiR-185 or the combination of both at half dose into CCl_4_-treated mice (Fig. 7a). Previously, we detected the agomiR-451 and agomiR-185 condensation of VLNPs and the VLNPs possessed outstanding gene condensed ability (Supplementary Fig. S6). The results of H&E staining showed that the combination of agomiR-451 and agomiR-185 induced fewer apoptotic hepatocytes and intralobular spotty necrosis around the vasculature than single agomir (Fig. 7b). Masson staining of collagen indicated that two agomirs in combination had better inhibitory effect on collagen deposition than individual agomir (Fig. 7c). In addition, the ALT and AST levels in CCl_4_-induced liver fibrosis mice were also significantly reduced by the agomir combination, suggesting their protective functions in liver fibrosis (Fig. 7d). Although agomiR-451/agomiR-185 alone showed limited effects on the hydroxyproline content in the livers of CCl_4_-treated mice, their combination induced a significant reduction in this liver damage marker, suggesting there was a collaborative function of these two agomirs (Fig. 7e). Moreover, agomiR-451 and agomiR-185 combination at half dose resulted in a more significant reduction in the protein expressions of EphB2 and other liver fibrogenesis-related markers including MMP2, α-SMA and TIMP2 (Fig. 7f, g). Further mechanisms study validated that overexpression of agomiR-451 (Fig. 7h) could inhibit the expression of XPO-1 (Fig. 7i, j) to upregulate the expression of mature miR-185 while had no effects on the expression of pri-miR-185 and pre-miR-185 in CCl_4_-treated mice (Fig. 7k). Taken together, all these results indicated that miR-451 combined with miR-185 could synergistically attenuate liver fibrosis partly via co-targeting EphB2, and the mechanism of synergy between these two miRNAs is associated with the contributory role of miR-451 in the post-transcriptional regulation of miR-185 maturation by targeting XPO-1.

## Discussion

Although the overexpression of EphB2 has been implicated in the development of hepatic fibrosis in mouse models^16^, the detailed cellular and molecular mechanisms for its upregulation in fibrosis have not been fully investigated. In view of aberrantly expressed miRNAs along with pathway analysis could provide a comprehensive understanding in the dysregulation of fibrogenic signaling components, two liver disease-related miRNAs including miR-451 and miR-185 were identified to investigate their modulating roles in the EphB2 expression. After examination of the EphB2 overexpression in immortalized HSC cell lines, isolated quiescent and activated primary HSCs, and fibrotic liver, we proved that EphB2 upregulation is a downstream molecular event of decreased expression of miR-451 and miR-185 in the process of liver fibrosis.

MiR-451 has been shown to exert various biological functions both in health condition and in pathogenesis of different diseases^27^. As far as liver disease is concerned, miR-451 was notably downregulated in HCC cells and tissues, and it could act as a tumor suppressor regulating hepatoma cell proliferation, invasion and migration^27^. A recent study demonstrated that miR-451 expression was downregulated in high-fat-diet-induced NASH mice, and that miR-451 regulates inflammatory cytokine production through the AMPK/AKT pathway via direct targeting Cab39 ^28^. Moreover, a significant downregulation of miR-451 was reported in liver of rats with nonalcoholic fatty liver disease by miRNA expression profiles analysis^29^. These prompted us to explore the role of miR-451 in liver fibrosis, and our results for the first time provided evidence that miR-451 is strongly decreased in activated HSCs and in mice with liver fibrosis.

Dysregulation of miR-185 expression has been reported in several types of fibrosis, such as idiopathic pulmonary fibrosis, renal fibrosis and liver fibrosis^30–33^. In most cases, miR-185 was decreased with the disease, but contradictory results were also reported. For example, elevated circulating miR-185 was reported in HBV-related liver fibrosis, which could target SREBF1 and increase expression of Col 1A1 and α-SMA^33^. A nonsignificant upregulation of miR-185 in HCC compared to normal tissues was revealed in all three datasets including TCGA, GSE10694 and GSE6857^34^. On the contrary, a recent research study with miRNA microarray analysis stated that miR-185 was significantly downregulated in activated HSCs induced by TGF-β1^35^. The expression of miR-185 was also shown downregulated by Illumina HiSeq sequencing in the plasma of patients with HBV-related liver fibrosis^32^. It was proposed that miR-185 inhibits activation of HSCs and prevents liver fibrosis via targeting the Ras homolog enriched in brain (RHEB) and the rapamycin-insensitive companion of mammalian target of rapamycin (RICTOR)^32^. Similarly, in our study, we found that miR-185 expression was decreased in activated HSCs and CCl_4_-induced liver fibrosis.

EphB2 was validated to be a target of both miR-451 and miR-185 from the following aspects. Firstly, bioinformatic analysis using available miRNA target-prediction strategies and tools online, such as miRBase and miRanda, indicated the presence of a putative target site for miR-451/miR-185 in the 3′UTR of *EphB2* mRNA. Secondly, overexpression of miR-451/miR-185 by synthetic mimics significantly inhibited, while downregulation of miR-451/miR-185 by synthetic inhibitor elevated the expression of EphB2 at protein level. Thirdly, luciferase reporter assay provided more convincing evidence that EphB2 is indeed a direct target of miR-451 and miR-185 in HSC cells.

Although different miRNAs may competitively bind the 3′UTR of the same gene, it was proposed that miRNAs might tend to synergistically control expression of target genes encoding extensively expressed proteins in human, which is more economical and efficient for gene regulation^36^. We then explored whether miR-451 and miR-185 could collaboratively act to fine-tune the protein output of EphB2. Our results showed that the combination of half-dose miR-451 and miR-185 mimics presented better inhibitory effect on the expression of EphB2 and other fibrotic markers than single miRNA mimics with full dose, suggesting that miR-451 and miR-185 play suppressing roles in liver fibrogenesis and there is a potent synergy between these two miRNAs.

During this research, a higher level of mature miR-185 rather than pri-miR-185 and pre-miR-185 was unexpectedly detected by miR-451 mimics treatment than the negative control treatment. We reasoned that the increase of miR-185 expression is possibly due to miR-451 targeting other genes of the microRNA biogenesis pathway in addition to EphB2. XPO-1 stood out as a good candidate due to its crucial roles in microRNA biogenesis and the presence of a conserved binding site of miR-451 on the 3′UTR region of its mRNA. The expression of XPO-1 was negatively correlated with miR-451 expression both in vitro and in vivo. The following luciferase reporter analysis confirmed XPO-1 as an additional target of miR-451. In addition, inhibition of the XPO-1 by a selective inhibitor verdinexor also increased the expression level of mature miR-185, indicating targeted inhibition of the nuclear transporter could restore fibrosis suppressive miRNA. However, the molecular factors linking XPO-1 and miR-185 expression still needs further study.

It is not a novel concept that liver fibrosis is a dynamic and potentially reversible process, but still no effective antifibrotic drugs are clinically available up to now. How to specifically and effectively deliver the antifibrotic drugs into activated HSCs has been widely explored^37,38^. Our group previously developed vitamin A-decorated biocompatible lipid nanoparticles for targeted delivery of dual-genes that could synergistically suppress collagen I accumulation in fibrogenesis^25^. In the present study, to evaluate the potential of miR-451/miR-185 in the treatment of liver fibrosis, miR-451/miR-185 agomirs or their combination were packaged within the same hyperbranched lipoid-based lipid nanoparticles (VLNPs) by our group. The employed vitamin A-decorated VLNPs displayed excellent gene condensed ability as well as transfection efficiency, and delivered precisely RNAs to HSCs. Correspondingly, in vivo administration of miR-451 and miR-185 agomir combination at half dose induced dual inhibition of EphB2 and improved antifibrosis efficiency than either monotherapy alone at full dose.

MiRNAs are known to target various genes at the post-transcriptional level. To examine the role of other targets in the therapeutic effect of miR-451/miR-185 agomirs, we detected the protein expression of three representative liver fibrogenesis-related target genes including *c-myc, RHEB* and *RICTOR* in CCl4-induced liver fibrosis mice. The transcription factor c-myc was identified as a common target of miR-451 and miR-185 using miRTarBase database. Overexpression of c-myc has been suggested as a novel marker of liver fibrosis in man and mice^39^. However, miR-451/miR-185 agomirs showed no effect on its expression in our study (Supplementary Fig. S7). RHEB and RICTOR were reported to mediate the effects of miR-185 on LX-2 cell activation^32^. Although our results showed that the expression of RHEB and RICTOR decreased slightly in some miR185 agomirs-treated mice, there is no significant difference in the expression of both proteins among all groups (Supplementary Fig. S7). The reasons for the discrepancy may be multifactorial and complex. The physiological and biochemical events of fibrosis may show differences between in vitro and in vivo cases. The inhibitory data obtained in vitro may be related to its more direct interactions on those targets. Besides, there were obvious individual variations in mice and our analysis was limited by the small sample sizes of the animals. Therefore, further studies with larger animal samples would be necessary to determine whether additional miR451/miR185 targets are involved in their antifibrotic activities.

**Fig. 8:**
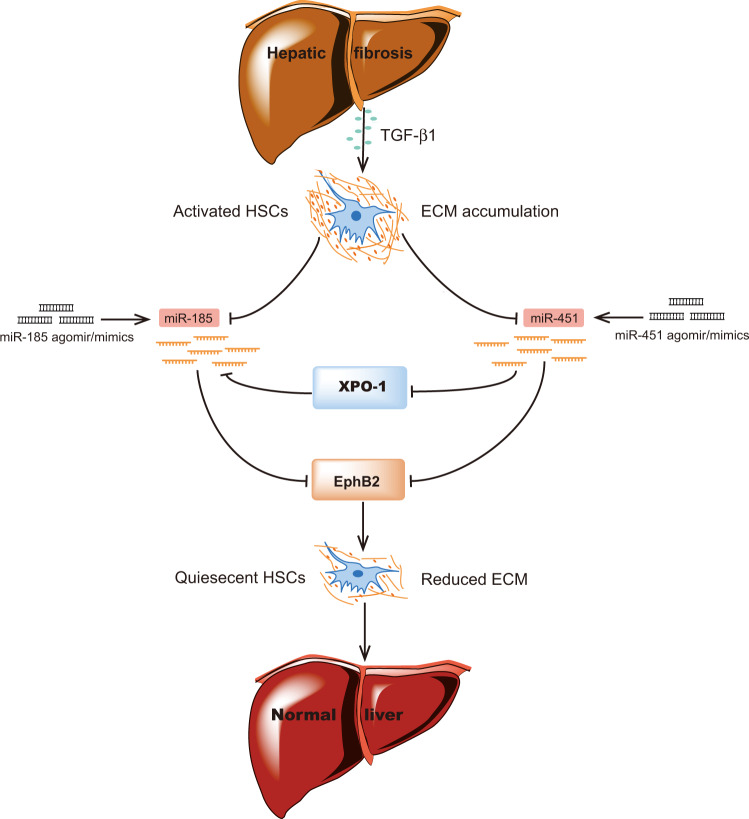
Schematic illustration. The possible pathways involved in the synergistic antifibrotic effects of miR-451 with miR-185.

In summary, our data have demonstrated that miR-451 and miR-185 function as fibrosis suppressors and act synergistically to inhibit liver fibrosis. Restoration of miR-451 and miR-185 at the same time prominently decreased EphB2 protein level, and suppressed liver fibrosis both in vitro and in vivo. The mechanism of their synergy is at least in part due to miR-451 also targeting XPO-1. As illustrated in Fig. 8, our findings not only provide new insights about the regulation of EphB2 overexpression in liver fibrosis, but also bring a novel miRNA-based therapeutic strategy for the treatment of hepatic fibrosis.

## Supplementary information

Supplementary Table 1

Supplementary Figure Legends

Supplementary Fig. S1

Supplementary Fig. S2

Supplementary Fig. S3

Supplementary Fig. S4

Supplementary Fig. S5

Supplementary Fig. S6

Supplementary Fig. S7
