# Supplementary material for: Synergistic antifibrotic effects of miR-451 with miR-185 partly by co-targeting EphB2 on hepatic stellate cells
Source: Cell Death Dis. 2020 May 28;11(5):402. doi: 10.1038/s41419-020-2613-y (PMC7256034; doi:10.1038/s41419-020-2613-y)
Supplement: Supplementary file 1 — Supplementary Table 1 [file 41419_2020_2613_MOESM1_ESM.docx]

**Supplementary Table 1. Primer sequences for RT-qPCR**

| **NO.** | **Genes** | **Forward** **primer sequence** | **Reverse primer sequence** |
| --- | --- | --- | --- |
| 1 | Human Col 1A1 | CCCAGAACATCACATATCAC | CAAGAGGAACACATATGGAG |
| 2 | Human α-SMA | GACAATGGCTCTGGGCTCTGTAA | CTGTGCTTCGTCACCCACGTA |
| 3 | Human GAPDH | GGATTTGGTCGTATTGGG | GGAAGATGGTGATGGGATT |
| 4 | Human MMP-2 | GTATTTGATGGCATCGCTCA | CATTCCCTGCAAAGAACACA |
| 5 | Human EphB2 | AAAAGGGCTTGGGAGATTCAT | GTCCATCTGTCCCGTCCTC |
| 6 | Human XPO-1 | ACGAGGAAGGAAGGAGCAGT | CGAGCTGCATGGTCTGCTAA |
| 7 | Mouse EphB2 | ACGCCACGGCCATAAAAAGCCC | TTGCCACTGTAGCGCCCATAGC |
| 8 | Mouse α-SMA | CGGGAGAAAATGACCCAGATT | AGGGACAGCACAGCCTGAATAG |
| 9 | Mouse MMP-2 | ACCTGAACACTTTCTATGGCTG | CTTCCGCATGGTCTCGATG |
| 10 | Mouse TIMP-2 | TCAGAGCCAAAGCAGTGAGC | GCCGTGTAGATAAACTCGATGTC |
| 11 | Mouse GAPDH | AGGTCGGTGTGAACGGATTTG | GGGGTCGTTGATGGCAACA |
| 12 | Mouse Col1A1 | GCTCCTCTTAGGGGCCACT | ATTGGGGACCCTTAGGCCAT |
| 13 | Mouse XPO-1 | AGCACTCATGGAGGCTCTTCA | GCCATGCGACTAACCATCAA |
| 14 | Rat Col1A1 | ATGTTCAGCTTTGTGGACCT | CAGCTGACTTCAGGGATGT |
| 15 | Rat α-SMA | TGTGCTGGACTCTGGAGATG | GAAGGAATAGCCACGCTCAG |
| 16 | Rat EphB2 | ATCGCCGTGGTCGTCATT | TTGGCAAACTCCCGCACT |
| 17 | Rat TIMP-2 | GCTGGACGTTGGAGGAAAGA | TGATGCTAAGCGTGTCCCAG |
| 18 | Rat XPO-1 | AGCACTCATGGAGGCTCTTCA | GCCATGCGACTAACCATCAA |
| 19 | Rat GAPDH | GAACCTGCCGTGGGTAGAG | AGGTCGGTGTGAACGGATTTG |
