# Supplementary material for: Synergistic antifibrotic effects of miR-451 with miR-185 partly by co-targeting EphB2 on hepatic stellate cells
Source: Cell Death Dis. 2020 May 28;11(5):402. doi: 10.1038/s41419-020-2613-y (PMC7256034; doi:10.1038/s41419-020-2613-y)
Supplement: Supplementary file 2 — Supplementary Figure Legends [file 41419_2020_2613_MOESM2_ESM.docx]

**Fig. S1 EphB2 is a target gene of miR-451 and miR-185 in HSC-T6 cells.**

(a) Expression of miR-451/miR-185 was examined by RT-qPCR in HSC-T6 cells transfected with corresponding miRNA mimics. (b) Western blotting analysis for EphB2 in HSC-T6 cells transfected with miR-451/miR-185 mimics. (c) Expression of miR-451/miR-185 was examined by RT-qPCR in HSC-T6 cells transfected with corresponding miRNA inhibitor. (d) Western blotting analysis for EphB2 in HSC-T6 cells transfected with miR-451/miR-185 inhibitor. Data are shown as the means ± SEM obtained from triplicate experiments (Student’s t-test, **P* < 0.05, ***P* < 0.01, and ****P* < 0.001).

**Fig. S2 MiR-451 and miR-185 inhibition promote activation of TGF-β1-induced HSCs.**

(a, c) Western blot analysis for MMP2, α-SMA and TIMP2 in LX-2 and HSC-T6 cells transfected with miR-451 inhibitor. (b, d) Western blot analysis for MMP2, α-SMA and TIMP2 in LX-2 and HSC-T6 cells transfected with miR-185 inhibitor. Data are shown as the means ± SEM obtained from triplicate experiments (Student’s t-test, **P* < 0.05, ***P* < 0.01, and ****P* < 0.001).

**Fig. S3 MiR-451 upregulates miR-185 at the post-transcriptional level by directly targeting XPO-1 in HSCs.**

(a) Effect of miR-451 overexpression on the expression of pri-miR-185, pre-miR-185, and mature miR-185 was detected by RT-qPCR in HSC-T6 cells. (b) RT-qPCR of miR-451 expression from LX-2 and HSC-T6 cells transfected with miR-185 mimics. (c, d) Western blotting analysis for XPO-1 in HSC-T6 cells with miR-451 overexpression. (e, f) Western blotting analysis for effect of verdinexor on the downregulation of XPO-1 in HSC-T6 cells. (g) The expression of pri-miR-185, pre-miR-185, and miR-185 was determined by RT-qPCR in HSC-T6 cells treated with verdinexor at dose of 0.5 or 1.0 μM. Data are shown as the means ± SEM obtained from triplicate experiments (Student’s *t*-test, **P* < 0.05, ***P* < 0.01, and ****P* < 0.001, n.s. nonsignificant).

**Fig. S4** **Expression of** **XPO-1 in activated HSC** **cell lines and CCl_4_-induced mice.**

(a, c) The protein expressions of XPO-1 were analyzed in LX-2 and HSC-T6 cells using western blotting, protein bands were quantified by Image J software. (b, d) The mRNA levels of *XPO-1* were examined in LX-2 and HSC-T6 cells using RT-qPCR. (e) The protein expression of XPO-1 was analyzed in CCl_4_-induced mice using western blotting, protein bands were quantified by Image J software (n=6 per group). (f) The mRNA expression of *XPO-1* was measured in CCl_4_-induced mice by RT-qPCR (n=6 per group). Data represent the means ± SEM obtained from triplicate experiments (Student’s *t*-test, **P* < 0.05, ** *P* < 0.01, and ****P* < 0.001).

**Fig. S5 Expression of EphB2 in activated HSCs** **treated with XPO-1 inhibitor.**

(a, c) Western blotting analysis of the expression of EphB2 in LX-2 and HSC-T6 cells treated with XPO-1 inhibitor, verdinexor. (b, d) The expression of EphB2 was determined by RT-qPCR in LX-2 and HSC-T6 cells treated with XPO-1 inhibitor, verdinexor. Data are shown as the means ± SEM obtained from triplicate experiments (Student’s *t*-test, **P* < 0.05 and ***P* < 0.01).

**Fig. S6 Agarose gel electrophoresis of VLNPs/miRNA at various weight ratios.**

(a) VLNPs/miR-185. (b) VLNPs/miR-451

**Fig. S7 Expressions of RICTOR, c-myc and RHEB in hepatic tissues after therapeutic administration of miR-451/185 agomirs.**

(a) Western blotting analysis for the expressions of RICTOR, c-myc and RHEB in hepatic tissues of representative mice from each group (n=6 per group), and (b) the intensity of the protein bands was quantified by Image J software. Data are shown as the means ± SEM (Student’s *t*-test, n.s. nonsignificant).
