# Supplementary figures and images for: Synergistic antifibrotic effects of miR-451 with miR-185 partly by co-targeting EphB2 on hepatic stellate cells
Source: Cell Death Dis. 2020 May 28;11(5):402. doi: 10.1038/s41419-020-2613-y (PMC7256034; doi:10.1038/s41419-020-2613-y)

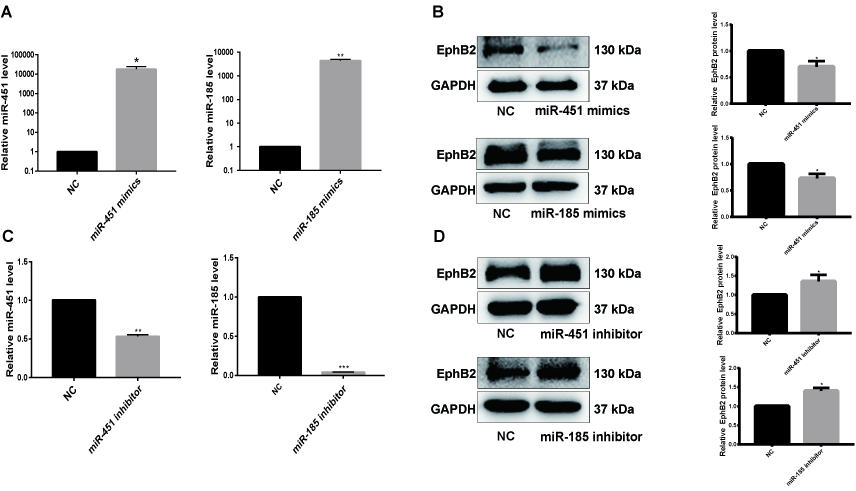

Supplement: Supplementary file 3 — Supplementary Fig. S1 [file 41419_2020_2613_MOESM3_ESM.tif]

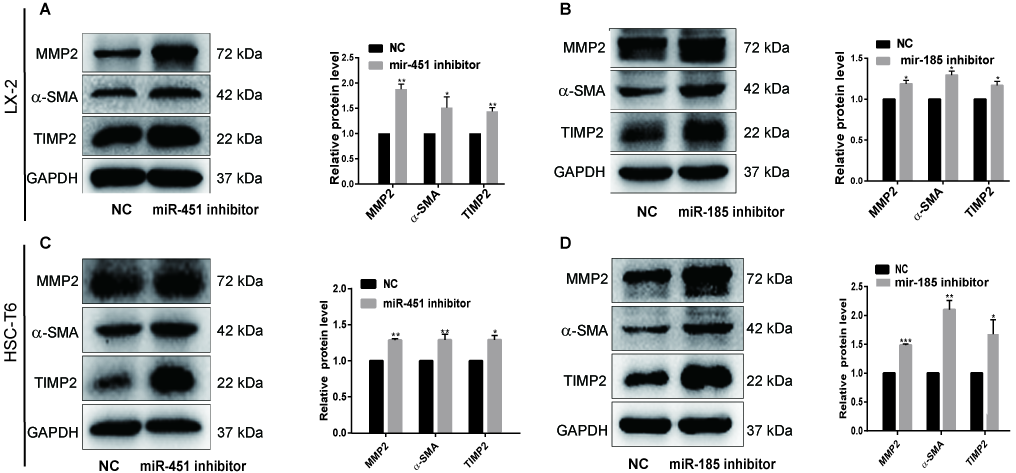

Supplement: Supplementary file 4 — Supplementary Fig. S2 [file 41419_2020_2613_MOESM4_ESM.tif]

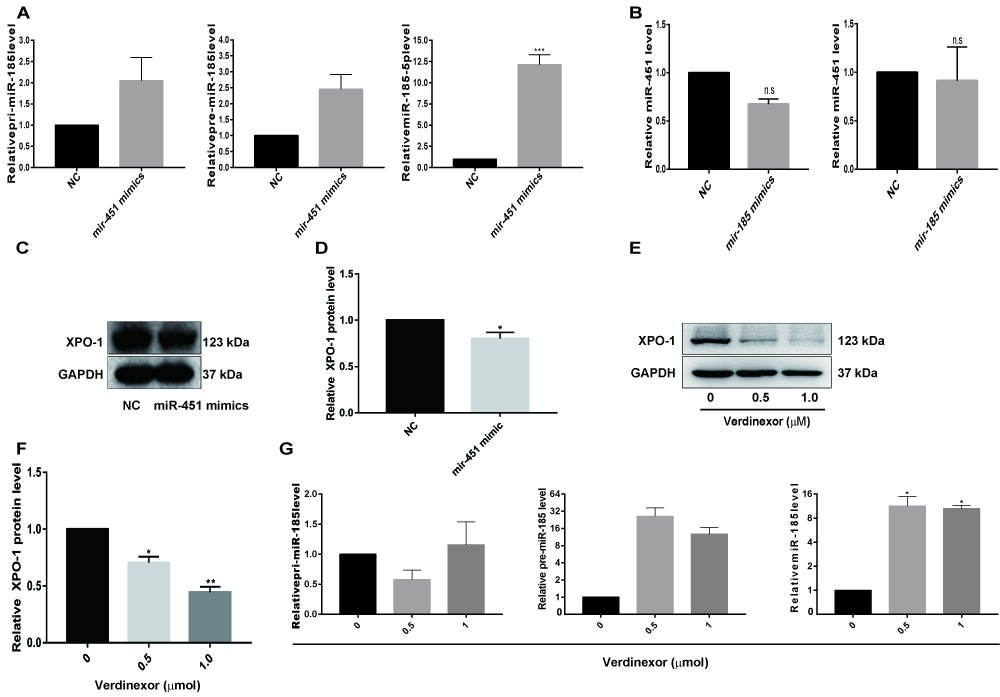

Supplement: Supplementary file 5 — Supplementary Fig. S3 [file 41419_2020_2613_MOESM5_ESM.tif]

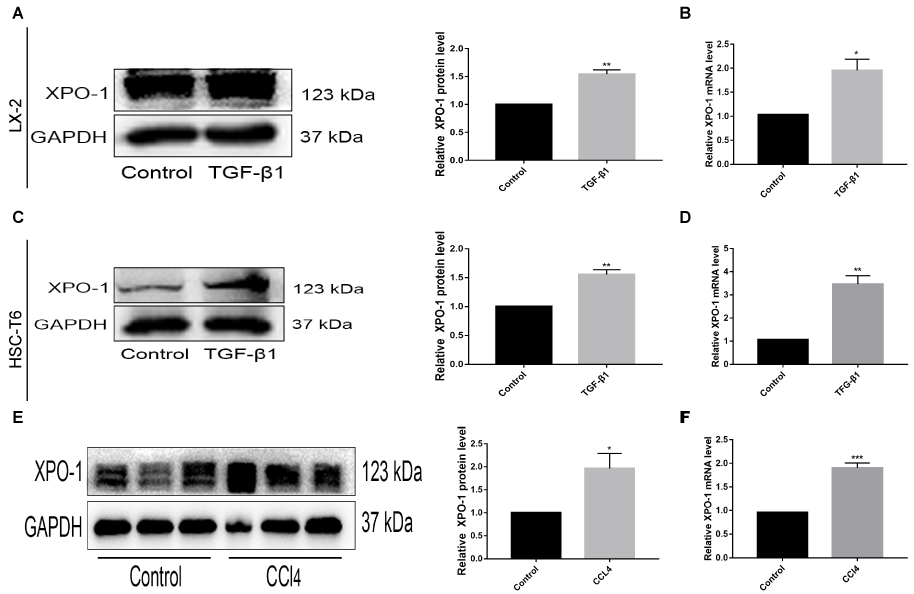

Supplement: Supplementary file 6 — Supplementary Fig. S4 [file 41419_2020_2613_MOESM6_ESM.tif]

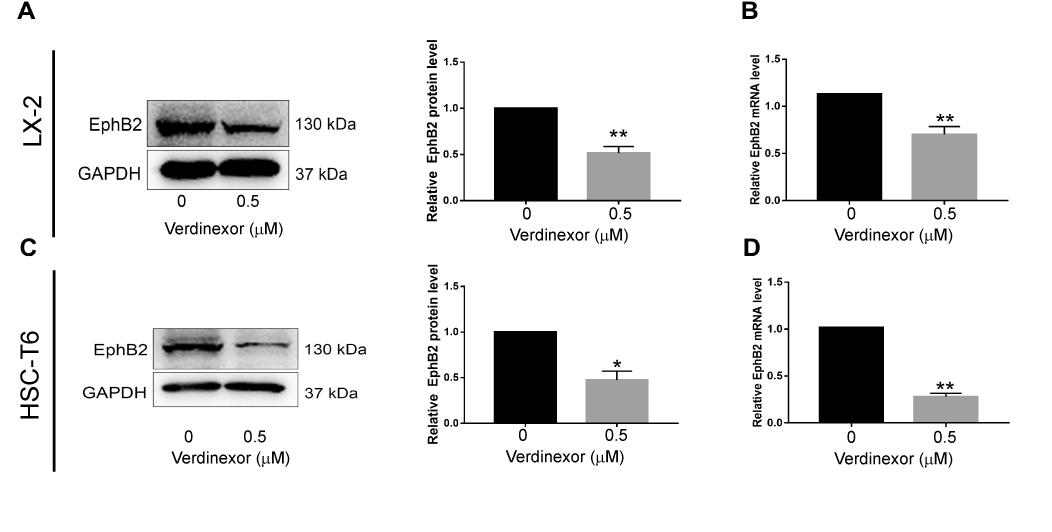

Supplement: Supplementary file 7 — Supplementary Fig. S5 [file 41419_2020_2613_MOESM7_ESM.tif]

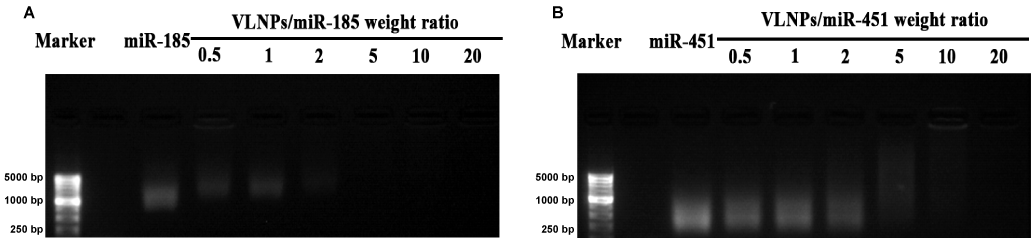

Supplement: Supplementary file 8 — Supplementary Fig. S6 [file 41419_2020_2613_MOESM8_ESM.tif]

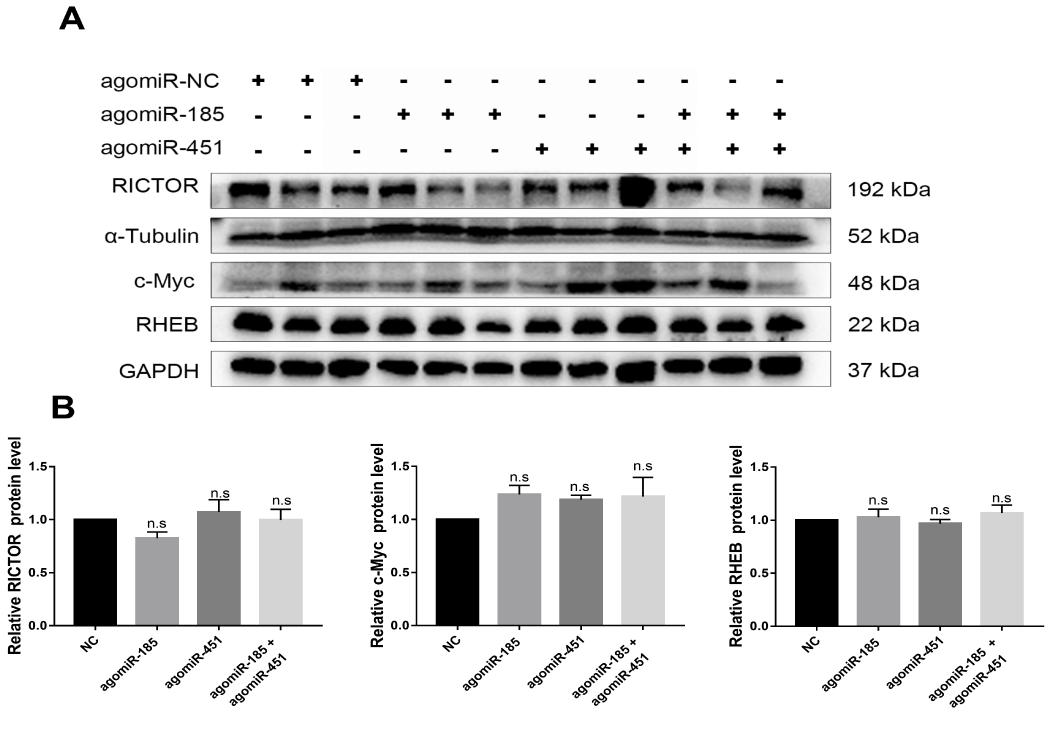

Supplement: Supplementary file 9 — Supplementary Fig. S7 [file 41419_2020_2613_MOESM9_ESM.tif]
